# Association Between Sex Hormones and Visual Field Progression in Women With Primary Open Angle Glaucoma: A Cross-Sectional and Prospective Cohort Study

**DOI:** 10.3389/fnagi.2021.756186

**Published:** 2021-12-24

**Authors:** Yichao Qiu, Jian Yu, Li Tang, Jun Ren, Mingxi Shao, Shengjie Li, Yunxiao Song, Wenjun Cao, Xinghuai Sun

**Affiliations:** ^1^Clinical Laboratory, Eye & ENT Hospital, Fudan University, Shanghai, China; ^2^Eye Institute and Department of Ophthalmology, Eye & ENT Hospital, Fudan University, Shanghai, China; ^3^Department of Clinical Laboratory, Shanghai Xuhui Central Hospital, Shanghai, China; ^4^NHC Key Laboratory of Myopia, Fudan University – Key Laboratory of Myopia, Chinese Academy of Medical Sciences, Shanghai, China; ^5^Shanghai Key Laboratory of Visual Impairment and Restoration, Shanghai, China

**Keywords:** primary open angle glaucoma (POAG), female, sex hormone, estrogen, visual field, cross-sectional study, cohort study

## Abstract

**Purpose:** We evaluated the level of sex hormones in female patients with primary open angle glaucoma (POAG) to determine whether they are associated with the onset and/or progression of POAG.

**Methods:** The cross-sectional study enrolled 63 women with POAG and 56 healthy women as normal control subjects. Furthermore, 57 women with POAG were included and followed-up for at least 2 years in the cohort study. All subjects were evaluated for serum concentration of sex hormones [prolactin (PRL), luteinizing hormone (LH), testosterone (TESTO), follicle-stimulating hormone (FSH), progesterone (PROG), and estrogen (E2)] and underwent visual field (VF) examination. In the cross-sectional study, Spearman analysis, linear regression analysis, and logistic regression analysis were performed to assess risk factors for POAG in women. In the cohort study, Cox regression analyses and Kaplan–Meier survival analysis were performed to identify factors associated with VF progression in women with POAG.

**Results:** In the cross-sectional study, the level of E2 was significantly lower in the POAG group than in the normal group (*p* < 0.05). Multiple logistic regression showed that the decreased level of E2 was a risk factor of POAG (OR = 0.27, 95% CI = 0.09–0.78, *p* < 0.05), especially in premenopausal subjects. In the cohort study, there were 29 non-progression subjects and 28 progression subjects. Patients in the progression group had significantly lower levels of E2 than those in the no progression group (*p* < 0.01). The decreased level of E2 at baseline was associated with POAG progression (HR = 0.08, 95% CI = 0.02–0.46, *p* < 0.05), especially in premenopausal subjects. Patients with POAG and with lower baseline E2 levels had significantly lower VF non-progression rates than patients with higher E2 levels (log-rank test *p* < 0.001), especially premenopausal subjects (log-rank test *p* < 0.05). Additionally, logistic regression analyses, Cox regression analyses, and Kaplan–Meier survival analysis showed that PROG, LH, FSH, and TESTO were risk factors of POAG and/or significantly associated with POAG progression.

**Conclusion:** A decreased E2 level is a POAG risk factor and is associated with VF progression in women with POAG, especially in premenopausal subjects. Additionally, other sex hormones (PROG, LH, FSH, and TESTO) might also play a role in POAG pathogenesis.

## Introduction

Primary glaucoma is the leading cause of irreversible visual field (VF) defect and blindness (Kingman, 2004). There were an estimated 79.6 million patients with glaucoma worldwide in 2020 (Quigley and Broman, 2006), and this number is expected to rise to 111.8 million by 2040 (Tham et al., 2014), with a disproportionate impact on people in Asia and Africa. The results of two surveys conducted in China showed that there is a considerable number of patients with primary open angle glaucoma (POAG) in China, and that the crude POAG prevalence is 2.6% (He et al., 2006; Wang et al., 2010).

Several risk factors have been proposed to affect the progression of glaucoma, including ethnicity (Kapetanakis et al., 2016; Kostanyan et al., 2016), age (Mukesh et al., 2002), genetic background (Stone et al., 1997; Rezaie et al., 2002; Thorleifsson et al., 2010), elevated intraocular pressure (IOP) (No authors listed, 2000; Gordon et al., 2002), vascular dysregulation (Leske, 2009), and thinner corneal thickness (Francis et al., 2008; Shin et al., 2015). Recently, women have been suspected to contribute to glaucoma progress, and sex hormones probably play an important role in glaucoma pathology (Tehrani, 2015; Nuzzi et al., 2019). Our previous study found that decreased estrogen (E2) is associated with a high risk of primary angle close glaucoma (PACG), and that the baseline E2 level can be used to predict the VF lost in postmenopausal women with PACG (Li S. et al., 2020). However, the contribution of sex hormones to the onset of POAG and VF progression remains uncertain.

There is some evidence of an association between a reduced risk of POAG and appropriately high E2 exposure, including during pregnancy, premenopause, and postmenopause with hormone therapy (Vajaranant and Pasquale, 2012; Dewundara et al., 2016). A Mayo clinic study that enrolled 1,044 women found that women with early menopause due to bilateral oophorectomy before the age of 43 have a 1.6 times higher risk of POAG (Vajaranant et al., 2014). In addition, randomized trials and observational studies have shown that postmenopausal women treated with E2 and progesterone (PROG) have a small but significantly decreased IOP (Uncu et al., 2006; Tint et al., 2010; Vajaranant et al., 2016) and reduced risk for POAG (Newman-Casey et al., 2014). Previous studies have used distinct psychological status and sex hormone treatment to indicate that the difference in sex hormone levels may be associated with the onset and/or development of glaucoma. Therefore, sex hormones should be taken into consideration as an important factor in the progression of functional damage.

We hypothesized that a low level of endogenous circulating sex hormones, especially E2, is associated with an increased risk of POAG and leads to a faster rate of VF loss. To test this hypothesis by determining the effects of sex hormones on the onset of POAG, we performed a cross-sectional study in women with POAG that assessed the levels of six sex hormones: prolactin (PRL), luteinizing hormone (LH), testosterone (TESTO), follicle-stimulating hormone (FSH), PROG, and E2. Furthermore, we performed a prospective cohort study to assess whether the baseline level of sex hormones was associated with VF progression in women with POAG.

## Materials and Methods

This study was conducted in the Department of Ophthalmology and Visual Sciences at the Eye and Ear, Nose, and Throat (ENT) Hospital of Fudan University, Shanghai, China. The study obtained Institutional Review Board/Ethics Committee approval from the Ethics Committee of the Eye and ENT Hospital, and adhered to the principles of the Declaration of Helsinki. Informed consent was obtained from the subjects.

### Subjects

In the cross-sectional study, female patients with POAG were recruited from the Department of Ophthalmology and Visual Sciences at the Eye and ENT Hospital of Fudan University from June 2016 to June 2018. During the study period, age-matched control group subjects were recruited from the population undergoing physical examination. The sex hormones and VF of the subjects with POAG were tested the first time they came to the hospital. The sex hormones and VF were tested on the same day. During the study period, the control subjects’ serum samples were evaluated on the day they came to the hospital to undergo a physical examination.

In the cohort study, female patients with POAG were recruited from the Department of Ophthalmology and Visual Sciences at the Eye and ENT Hospital of Fudan University from June 2016 to June 2018, and were followed up for at least 2 years. The relationships between POAG and sex hormones and VF progression were assessed in premenopausal and postmenopausal female patients with POAG, respectively. All subjects in the POAG group received IOP-lowering medication, and none had surgical treatment during the follow-up. The VF in all patients with POAG was tested the first time they came to the hospital and during each return visit (6, 12, 18, and 24 months after the first visit). The sex hormones were evaluated the first time they came to the hospital. We evaluated the sex hormone levels of POAG subjects before they received POAG treatment in our hospital.

### Diagnostic and Inclusion Criteria

All POAG diagnoses were determined by a glaucoma specialist. The POAG diagnostic criteria, described previously (Li et al., 2018, 2019b), included high IOP (>21 mmHg), open anterior chamber angles on gonioscopy, typical glaucomatous optic disk neuropathy, and glaucomatous VF defects. VF defect was defined as three or more non-edge contiguous points in the same hemifield, the probability of which is <5% in age-matched control groups (one of which was <1%), and the abnormal standard deviation pattern of occurrence in the normal population, which has a *p*-value < 0.05. It met the following test reliability criteria: <20% fixation losses, <15% false positives, or <15% false negatives.

All subjects met the following inclusion criteria (Li et al., 2021): female; no secondary glaucoma or any other eye diseases with visual acuity or VF deficiency; no intraocular surgery 2 months before recruitment into our study; no systemic diseases, including autoimmune disease, metabolic syndrome, acute infectious disease, or cancer; and not taking drugs that could affect sex hormone levels.

**FIGURE 1:**
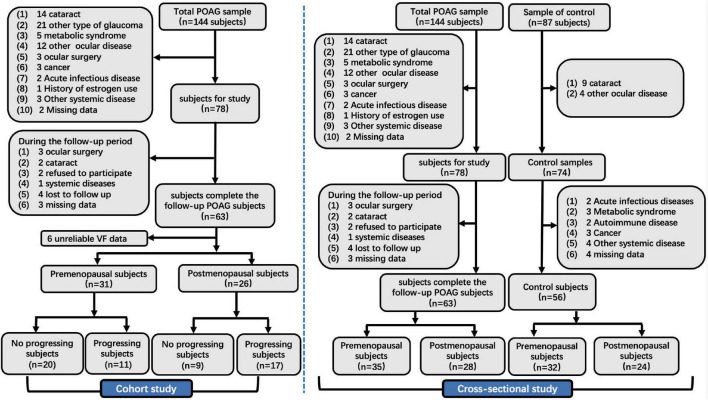
The flow chart of the cross-sectional and cohort study. POAG, primary open angle glaucoma; IOP, intraocular pressure; VCDR, vertical cup-to-disk ratio; VF, visual field.

In the cross-sectional study, we identified 144 patients who met the POAG diagnostic criteria, and 66 patients were ruled out based on the inclusion criteria. Meanwhile, 87 healthy subjects were recruited from physical examination, and 31 normal subjects were excluded based on the inclusion criteria. Finally, 63 female patients with POAG (35 premenopausal and 28 postmenopausal) and 56 normal control subjects (32 premenopausal and 24 postmenopausal) were enrolled in our cross-sectional study (Figure 1).

In the cohort study, we identified 144 patients who met the POAG diagnostic criteria. Of this population, 87 patients were ruled out based on the inclusion criteria. Finally, we analyzed VF data from 57 patients with POAG in our cohort study, including 31 premenopausal and 26 postmenopausal subjects (Figure 1).

### Examination

The clinical data of the patients with POAG were collected by a glaucoma specialist during a standardized ophthalmic examination. All IOP measurements were taken by one skilled ophthalmologist. All subjects had their IOP measured before receiving POAG treatment at the hospital. IOP was tested three times using Goldmann applanation tonometry (GAT; Haag-Streit, Bern, Switzerland), and then an average value was calculated. Gonioscopy was used to assess the anterior chamber angle. The central corneal thickness (CCT), axial length (AL), and anterior chamber depth (ACD) were measured using A-scan ultrasound (A-Scan Pachymeter, Ultrasonic, Exton, PA, United States). The fundus was analyzed using a digital retinal camera (TRC-NW200, Topcon Medical Systems, Oakland, NJ, United States), and two doctors evaluated the vertical cup-to-disk ratio (VCDR) based on the fundus photographs and then took the average values. In the normal control subjects, slit-lamp and gonioscopy were used to confirm that the subjects were free of glaucoma; other ophthalmic examinations (IOP, CCT, AL, ACD, VCDR) were not performed.

Specialty physicians from the Eye and ENT Hospital of Fudan University conducted the medical examinations of all subjects, which included the assessment of height, weight, heart rate, blood pressure, blood glucose, body temperature, electrocardiogram, radiograph, liver function, renal function, infectious disease, and IOP-lowering medication. The body mass index (BMI) was calculated as the weight in kilograms divided by the height in meters squared.

Laboratory examination was performed by the Departments of Clinical Laboratory of the Eye and ENT Hospital. The blood samples of the POAG and normal subjects were collected from the 14th to the 28th day of the menstrual cycle (secretory phase). Blood samples were taken *via* standard venipuncture to test the levels of sex hormones (PRL, LH, TESTO, FSH, PROG, and E2). All sample tubes were centrifuged for 10 min at 3,000 rpm, and it took less than 3 h to complete the entire process from collection to testing. We used a commercially available kit (Roche Diagnostics GmbH, Mannheim, Germany) for sensitive electrochemiluminescence detection to measure the sex hormone levels. During the last 5-year study period, internal controls were analyzed daily, and the variation coefficients varied from 2.1% to 3.8%, with no significant changes in the value.

### Visual Field Analysis

The methods used to analyze VF were previously described (Li et al., 2019a; Li S. et al., 2020). After excluding eye infection and no light perception, all patients with POAG underwent VF examination at the Glaucoma Department of the Eye and ENT Hospital of Fudan University. The VF mean deviation (MD) and determined using Octopus perimeter (G1 program, Haag-Streit, Bern, Switzerland) were taken into consideration for statistical analysis. All patients with POAG took the VF test at least three times. After excluding the first two tests, the ophthalmologists chose one reliable VF result (false positive/negative value < 15% and reliability factor value < 20%) and one compatible VF result to record. VF testing was performed initially when a patient was recruited and every 6 months during the follow-up period. Each subject met the following criteria: the number of reliable VF results was ≥5 and the follow-up period were >2 years. Functional damage progression was defined by meeting one of the following criteria during Humphrey perimetry testing: (Naghizadeh and Holló, 2014; Chen et al., 2018) (1) developing a new scotoma of ≥3 non-edge points worsening ≥5 dB, or one non-edge point worsening ≥10 dB; (2) a cluster of ≥3 non-edge points with ≥10 dB deterioration in a preexisting scotoma; (3) developing a new cluster of ≥3 non-edge points with 15° around a preexisting scotoma; and (4) worsening of the global MD value by ≥2 dB per year. The eyes with POAG were selected for observation during the follow-up period. If the patients had bilateral POAG, we randomly chose one eye to be involved in our study.

### Statistical Analysis

SPSS software (version 26; SPSS Inc., Chicago, IL, United States) was used for statistical analyses. GraphPad Prism (version 8.4; La Jolla, CA, United States) was used to create the figures. A sample size calculation was undertaken to determine the study’s recruitment sample sizes. We used an open-source calculator to calculate the minimal required sample size based on an odds ratio (OR) = 6, α = 0.05, β = 0.20, and a prevalence of POAG of about 5%. The obtained sample size for each group was 54 for the cross-sectional study. In the cohort study, assuming a VF endpoint in 45% of patients by the end of 2 years, the hazard ratio (HR) was 3. Fixing α = 0.05 and β = 0.20, the mean progress time was 12 months in the progression group, and 58 patients were needed for the analysis.

Depending on the data type, mean ± standard deviation (SD) or percentage were used to describe the results. The Kolmogorov–Smirnoff test was used to estimate normality. Based on data type and distribution, the independent student *t*, Mann–Whitney *U*, Fisher’s exact, and χ^2^ test were used to compare groups. Spearman analysis was used to analyze the correlations between sex hormones and ocular parameters. Then, univariate linear regressions and multivariate linear regression were used to assess the associations between sex hormones and MD. Age, BMI, diastolic blood pressure (DBP), diabetes (yes = 1, no = 0), and hypertension (yes = 1, no = 0) were adjusted for in multivariate linear regression. In consideration of the data distribution of sex hormone levels, the log10 (Lg) transformation of sex hormones was carried out, and linear regression analysis was performed. Univariate and multivariate logistic regression analyses were used to assess the association between sex hormones and the risk of POAG in the premenopausal and postmenopausal subgroups, respectively. Moreover, multivariate logistic regression was adjusted for age, BMI, diabetes (yes = 1, no = 0), and hypertension (yes = 1, no = 0). The logistic regression results were presented as OR and corresponding 95% confidence intervals (CIs). A two-side *p*-value of <0.05 was considered statistically significant.

In the cohort study, patients with POAG were divided into premenopausal and postmenopausal subgroups. The subjects in each subgroup were then divided into a progression group and a no progression group based on the MD growth rate. The association between sex hormones and POAG progression was tested by univariate and multivariate Cox proportional hazards analyses, which was adjusted for age, BMI, DBP, systolic blood pressure (SBP), diabetes (yes = 1, no = 0), and hypertension (yes = 1, no = 0). Based on the median E2 level, patients with POAG were divided into subgroups of E2 < 45.1 and E2 > 45.1. Kaplan–Meier survival analysis was used to assess survival outcomes, and the log-rank estimated the association between sex hormones and POAG progression in female, further in premenopausal and postmenopausal subgroups. A two-sided *p*-value of <0.05 was considered statistically significant.

## Results

### Cross-Sectional Study

A total of 63 female patients with POAG and 56 female normal subjects were enrolled in this study. Table 1 compares the characteristics of the two groups, including age, BMI, presence of diabetes and hypertension, and FSH, PROG, PRL, TESTO, LH, and E2 levels. The E2 level was significantly lower (*p* < 0.05) in the POAG group (136.24 ± 193.47 pg/mL) than in the normal group (235.89 ± 316.10 pg/mL). The POAG subjects also had significantly lower E2 levels than the normal subjects in the premenopausal subgroup (223.03 ± 224.93 pg/mL, 389.30 ± 347.02 pg/mL, *p* = 0.02, respectively) and in the postmenopausal subgroup (27.76 ± 16.63 pg/mL, 31.34 ± 15.14 pg/mL, *p* = 0.03, respectively).

**TABLE 1:** Comparison of characteristics in women with POAG and normal subjects.

Variable	Control group (*n* = 56)	POAG group (*n* = 63)	*t*-Value	*P*-Value
Age (year), mean ± SD	52.52 ± 11.61	52.76 ± 18.49	0.09	0.93^a^
BMI (kg/m^2^), mean ± SD	22.20 ± 2.69	21.24 ± 3.08	1.78	0.08^a^
Diabetes, *n* (%)	1 (1.8%)	3 (4.8%)	0.14	0.71^b^
Hypertension, *n* (%)	11 (19.6%)	18 (28.1%)	1.17	0.28^b^
Topical glaucoma medications, *n* (%)				
0–2		23 (36.5%)		
>2		40 (63.5%)		
IOP (mmHg), mean ± SD		21.75 ± 7.86		
VCDR, mean ± SD		0.76 ± 0.19		
CCT (mm), mean ± SD		536.25 ± 38.46		
ACD (cm), mean ± SD		2.73 ± 0.65		
AL (cm), mean ± SD		25.33 ± 3.17		
MD (dB), mean ± SD		13.45 ± 8.69		
MS (dB), mean ± SD		13.78 ± 9.22		
PRL (ng/mL), mean ± SD	356.72 ± 275.16	369.47 ± 381.78	−0.47	0.64^c^
Premenopausal	455.76 ± 320.15	481.79 ± 478.93	−0.74	0.46^c^
Postmenopausal	224.68 ± 105.19	229.08 ± 95.21	−0.42	0.67^c^
LH (mIU/mL), mean ± SD	21.99 ± 15.85	21.55 ± 15.74	−0.23	0.82^c^
Premenopausal	14.52 ± 15.25	12.48 ± 12.57	−0.46	0.64^c^
Postmenopausal	31.97 ± 10.28	32.89 ± 11.41	−0.29	0.78^c^
TESTO (ng/mL), mean ± SD	0.71 ± 0.41	1.06 ± 1.26	−1.05	0.29^c^
Premenopausal	0.88 ± 0.41	1.20 ± 1.03	−0.89	0.38^c^
Postmenopausal	0.48 ± 0.28	0.88 ± 1.49	−0.55	0.58^c^
FSH (mIU/mL), mean ± SD	30.39 ± 33.29	49.84 ± 41.24	−1.16	0.25^c^
Premenopausal	19.89 ± 27.66	24.30 ± 32.12	−0.17	0.87^c^
Postmenopausal	65.38 ± 19.63	81.75 ± 26.53	−2.44	**0.02^c^**
PROG (ng/mL), mean ± SD	6.65 ± 14.24	4.36 ± 10.96	−1.20	0.23*^c^*
Premenopausal	10.81 ± 17.82	7.31 ± 14.09	−0.14	0.89^c^
Postmenopausal	1.11 ± 0.75	0.67 ± 0.31	−2.94	0.01^c^
E2 (pg/mL), mean ± SD	235.89 ± 316.10	136.24 ± 193.47	−2.20	**0.03^c^**
Premenopausal	389.30 ± 347.02	223.03 ± 224.93	−2.42	**0.02^c^**
Postmenopausal	31.34 ± 15.14	27.76 ± 16.63	−2.22	**0.03^c^**

*^a^Independent samples t-test.*

*^b^χ^2^ test.*

*^c^Mann–Whitney U test.*

*POAG, primary open angle glaucoma; SD, standard deviation; IOP, intraocular pressure; VCDR, vertical cup-to-disk ratio; AL, axial length; CCT, central corneal thickness; ACD, anterior chamber depth; MD, mean deviation; MS, mean sensibility. PRL, prolactin; LH, luteinizing hormone; TESTO, testosterone; FSH, follicle-stimulating hormone; PROG, progesterone; E2, 17-β-estradiol. Bold values highlight the P value of <0.05.*

The associations between sex hormones and ocular parameters were assessed using Spearman analysis (Supplementary Figure 1). PRL level was significantly positively associated with ACD. Except for PRL, all measured sex hormones were significantly negatively correlated with IOP (*p* < 0.05). Both PROG and E2 had a statistically significant negative correlation with MD (*R* = −0.48, *p* < 0.01; *R* = −0.33, *p* = 0.01, respectively).

The linear regression results for demographic parameters and sex hormones with MD in female patients with POAG are shown in Table 2. Univariate linear regression showed that there was a significant negative correlation between E2 and MD (*B* = −0.04, *p* < 0.01, 95% CI = −0.06 to 0.02) and between Lg (E2) and MD (*B* = −8.60, *p* < 0.01, 95% CI = −13.40 to −3.81). After adjusting for age, diabetes (yes = 1, no = 0), hypertension (yes = 1, no = 0), DBP, and BMI, E2 (*B* = −0.05, *p* < 0.01, 95% CI = −0.09 to −0.02) and Lg (E2) (*B* = −14.19, *p* < 0.01, 95% CI = −23.30 to −5.10) also showed statistically negative correlations with MD.

**TABLE 2:** Univariate linear regressions and multiple linear regressions for associations between characteristics and blood sex hormone level with visual field mean deviation and in women with POAG.

Variable	*B*	*t*-Value	*p*-Value	95% CI
**Model A**				
Age	0.14	2.23	**0.03**	0.02 to 0.27
Diabetes	0.48	0.09	0.93	−9.95 to 10.90
Hypertension	2.08	0.81	0.42	−3.07 to 7.23
BMI	0.17	0.46	0.64	−0.57 to 0.91
PRL	0.00	0.09	0.93	−0.01 to 0.01
LH	0.07	0.93	0.36	−0.08 to 0.22
TESTO	−0.61	−0.68	0.50	−2.40 to 1.18
FSH	0.04	1.30	0.20	−0.02 to 0.10
PROG	−0.02	−0.13	0.90	−0.24 to 0.21
E2	−0.04	−3.77	**<0.01**	−0.06 to 0.02
Lg (PRL)	−2.08	−0.56	0.58	−9.55 to 5.38
Lg (LH)	2.87	1.07	0.29	−2.52 to 8.26
Lg (TESTO)	−4.93	−1.67	0.10	−10.85 to 0.99
Lg (FSH)	3.48	1.82	0.07	−0.35 to 7.31
Lg (PROG)	−5.26	−2.47	**0.02**	−9.54 to −0.99
Lg (E2)	−8.60	−3.60	**<0.01**	−13.40 to −3.81
**Model B**				
PRL	<0.01	0.51	0.61	−0.01 to 0.01
LH	−0.07	−0.60	0.55	−0.28 to 0.15
TESTO	−0.03	−0.03	0.98	−1.96 to 1.90
FSH	−0.03	−0.67	0.51	−0.14 to 0.07
PROG	0.09	0.75	0.46	−0.15 to 0.33
E2	−0.05	−3.09	**<0.01**	−0.09 to −0.02
Lg (PRL)	−0.05	−0.01	0.99	−8.70 to 8.61
Lg (LH)	−1.74	−0.43	0.67	−9.85 to 6.36
Lg (TESTO)	−1.77	−0.46	0.65	−9.54 to 5.99
Lg (FSH)	0.12	0.03	0.98	−7.95 to 8.19
Lg (PROG)	−4.08	−1.50	0.14	−9.54 to 1.38
Lg (E2)	−14.19	−3.14	**<0.01**	−23.30 to −5.10

*Model A is not adjusted. Model B adjusted for age, BMI, DBP, diabetes (yes = 1, no = 0), hypertension (yes = 1, no = 0).*

*POAG, primary open angle glaucoma; B, regression coefficient; CI, confidence interval; BMI, body mass index; PRL, prolactin; LH, luteinizing hormone; TESTO, testosterone; FSH, follicle-stimulating hormone; PROG, progesterone; E2, 17-β-estradiol; Lg (), values after log 10 transformation; SBP, systolic blood pressure; DBP, diastolic blood pressure. Bold values highlight the P value of <0.05.*

Univariate and multivariate logistic regression analyses were performed to identify the risk factors for female patients with POAG (Table 3). Multivariate logistic regression analyses demonstrated a significant association between E2 and POAG (OR = 0.99, 95% CI = 0.99–1.00, *p* = 0.03). After Lg transformation, E2 also showed a significant association with POAG (OR = 0.27, 95% CI = 0.09–0.78, *p* = 0.02).

**TABLE 3:** Univariate and multivariate logistic regression analyses to identify risk factors for women with POAG.

	Univariate		Multivariate	
	*P*-value	OR (95% CI)	*P*-value	OR (95% CI)
Age	0.93	1.00 (0.98 to 1.03)		
Diabetes	0.39	2.75 (0.28 to 27.23)		
Hypertension	0.26	1.64 (0.70 to 3.85)		
BMI	0.08	0.89 (0.78 to 1.01)		
PRL	0.84	1.00 (0.99 to 1.00)	0.54	1.00 (0.99 to 1.00)
LH	0.88	1.00 (0.98 to 1.02)	0.63	0.99 (0.96 to 1.02)
TESTO	0.07	1.82 (0.95 to 3.49)	0.07	2.14 (0.93 to 4.93)
FSH	0.13	1.01 (1.00 to1.02)	0.07	1.01 (1.00 to 1.03)
PROG	0.33	0.99 (0.96 to 1.02)	0.50	0.99 (0.96 to 1.02)
E2	**0.048**	0.998(0.997 to 1.00)	**0.03**	0.99 (0.99 to 1.00)
Lg (PRL)	0.81	0.86 (0.25 to 2.94)	0.95	0.96 (0.24 to 3.82)
Lg (LH)	0.77	0.88 (0.38 to 2.06)	0.49	0.67 (0.21 to 2.13)
Lg (TESTO)	0.15	2.13 (0.77 to 5.89)	0.08	3.10 (0.88 to 10.92)
Lg (FSH)	0.52	1.23 (0.66 to 2.29)	0.48	1.43 (0.53 to 3.86)
Lg (PROG)	0.22	0.69 (0.38 to 1.26)	0.28	0.67 (0.33 to 1.37)
Lg (E2)	0.07	0.55 (0.29 to 1.04)	**0.02**	0.27 (0.09 to 0.78)

*Multivariate logistic regression was adjusted for age, BMI, diabetes (yes = 1, no = 0), hypertension (yes = 1, no = 0).*

*POAG, primary open angle glaucoma; OR, odds ratio; CI, confidence interval; BMI, body mass index; PRL, prolactin; LH, luteinizing hormone; TESTO, testosterone; FSH, follicle-stimulating hormone; PROG, progesterone; E2, 17-β-estradiol; Lg (), values after log 10 transformation. Bold values highlight the P value of <0.05.*

Univariate and multivariate logistic regression analyses were also performed with data from the premenopausal (Supplementary Table 1) and postmenopausal subgroups (Supplementary Table 2). In the premenopausal group, multivariate logistic regression analysis revealed a statistical association between E2 and POAG (OR = 0.09, 95% CI = 0.02–0.45, *p* = 0.03). In the postmenopausal subgroup, TESTO and PROG showed a significant association with the risk of POAG (OR = 9.89, 95% CI = 1.26–77.65, *p* = 0.03; OR = 0.02, 95% CI = 0.00–0.75, *p* = 0.04, respectively).

### Cohort Study

According to the criteria for functional VF progression, patients with POAG were divided into a progression group (*n* = 28) and a no progression group (*n* = 29). The demographic characteristics, ocular parameters, and sex hormone levels of the two groups at baseline are summarized in Table 4. Patients in the progression group had significantly lower levels of E2 (26.99 ± 51.69 pg/mL) than those in the no progression group (211.26 ± 242.20 pg/mL) (*p* < 0.01).

**TABLE 4:** Comparison of characteristics of no progression group and progression group in POAG subjects.

Variable	No progression (*n* = 29)	Progression (*n* = 28)	*t*-Value	*P*-Value
Age (year), mean ± SD	46.93 ± 17.16	59.00 ± 17.69	−2.61	**0.01^a^**
BMI (kg/m^2^), mean ± SD	21.53 ± 2.64	20.87 ± 3.66	0.78	0.44^a^
SBP (mmHg), mean ± SD	123.52 ± 19.08	127.61 ± 18.71	−0.82	0.42^a^
DBP (mmHg), mean ± SD	70.55 ± 8.07	73.07 ± 8.50	−1.15	0.26^a^
Diabetes, *n* (%)	0(0%)	3(10.7%)	3.28	0.11^b^
Hypertension, *n* (%)	5(17.2%)	18(64.3%)	13.10	**<0.01^b^**
Topical glaucoma medications				
0–2	9(31.0%)	10(35.7%)		
>2	20(69.0%)	18(64.3%)	0.14	0.71^b^
IOP (mmHg), mean ± SD	23.13 ± 9.11	22.36 ± 9.79	−0.94	0.35^c^
VCDR, mean ± SD	0.76 ± 0.19	0.78 ± 0.19	−0.45	0.66^a^
CCT (mm), mean ± SD	533.28 ± 37.70	538.46 ± 40.35	−0.47	0.64^a^
ACD (cm), mean ± SD	2.70 ± 0.66	2.78 ± 0.66	−0.40	0.69^a^
AL (cm), mean ± SD	25.17 ± 2.45	25.42 ± 3.95	−0.27	0.79^a^
MD (dB), mean ± SD	11.83 ± 9.69	15.12 ± 7.32	−1.35	0.18^c^
MS (dB), mean ± SD	15.58 ± 10.41	12.34 ± 7.51	−1.35	0.28^c^
PRL (ng/mL), mean ± SD	366.11 ± 342.23	378.27 ± 459.90	−0.11	0.91^a^
LH (mIU/mL), mean ± SD	18.68 ± 16.90	24.12 ± 13.18	−1.35	0.18^a^
TESTO (ng/mL), mean ± SD	1.34 ± 1.11	0.80 ± 1.46	−3.89	**<0.01^c^**
FSH (mIU/mL), mean ± SD	38.57 ± 45.52	60.34 ± 30.39	−2.24	**0.03^c^**
PROG (ng/mL), mean ± SD	6.83 ± 14.17	0.71 ± 0.48	−4.21	**<0.01^c^**
E2 (pg/mL), mean ± SD	211.26 ± 242.20	26.99 ± 51.69	−3.54	**<0.01^c^**

*^a^Independent samples t-test.*

*^b^χ^2^ test.*

*^c^Mann–Whitney U test.*

*POAG, primary open angle glaucoma; SD, standard deviation; BMI, body mass index; SBP, systolic blood pressure; DBP, diastolic blood pressure; IOP, intraocular pressure; VCDR, vertical cup-to-disk ratio; CCT, central corneal thickness; ACD, anterior chamber depth; AL, axial length; MD, mean deviation; MS, mean sensibility; PRL, prolactin; LH, luteinizing hormone; TESTO, testosterone; FSH, follicle-stimulating hormone; PROG, progesterone; E2, 17-β-estradiol. Bold values highlight the P value of <0.05.*

Univariate and multivariate Cox proportional hazards regression analyses were performed to assess the associations between baseline parameters and POAG progression (Table 5). After adjusting for age, BMI, DBP, SBP, diabetes, and hypertension, patients with lower baseline E2 levels had a significantly higher risk of POAG progression (HR = 0.99, 95% CI = 0.98–1.00, *p* = 0.02). After log10 transformation, E2 was found to have similar results in multivariate Cox proportional hazards regression analysis (HR = 0.08, 95% CI = 0.02–0.46, *p* < 0.01).

**TABLE 5:** Univariate Cox proportional hazards regression analysis and multivariate Cox proportional hazards regression analysis to assess the value of baseline parameters associated with progression of POAG.

	Univariate		Multivariate	
	*P*	HR (95% CI)	*P*	HR (95% CI)
Age	**0.04**	1.02 (1.00 to 1.05)		
Diabetes	**0.02**	4.39 (1.27 to 15.19)		
Hypertension	0.33	1.47 (0.68 to 3.18)		
BMI	0.46	0.96 (0.85 to 1.07)		
SBP	0.69	1.00 (0.99 to 1.02)		
DBP	0.31	1.02 (0.98 to 1.07)		
PRL	0.97	1.00 (1.00 to 1.00)	0.52	1.00 (1.00 to 1.00)
LH	0.35	1.01 (0.99 to 1.04)	0.39	0.98 (0.95 to 1.02)
TESTO	0.18	0.67 (0.37 to 1.21)	0.75	0.93 (0.57 to 1.51)
FSH	0.14	1.01 (1.00 to 1.02)	0.94	1.00 (0.99 to 1.01)
PROG	**0.05**	0.34 (0.12 to 1.01)	0.10	0.38 (0.12 to 1.20)
E2	**0.02**	0.99 (0.99 to 1.00)	**0.02**	0.99 (0.98 to 1.00)
Lg (PRL)	0.74	0.82 (0.24 to 2.73)	0.68	1.33 (0.34 to 5.19)
Lg (LH)	0.16	1.99 (0.76 to 5.23)	0.78	0.82 (0.21 to 3.26)
Lg (TESTO)	**<0.01**	0.22 (0.08 to 0.61)	0.22	0.41 (0.10 to 1.72)
Lg (FSH)	**0.02**	2.45 (1.16 to 5.18)	0.15	2.39 (0.73 to 7.84)
Lg (PROG)	**<0.01**	0.08 (0.02 to 0.43)	**<0.01**	0.08 (0.01 to 0.52)
Lg (E2)	**<0.01**	0.20 (0.07 to 0.57)	**<0.01**	0.08 (0.02 to 0.46)

*Multivariate Cox regression was adjusted for age, BMI, DBP, SBP, diabetes (yes = 1, no = 0), hypertension (yes = 1, no = 0).*

*POAG, primary open angle glaucoma; HR, hazard ratio; CI, confidence interval; BMI, body mass index; PRL, prolactin; LH, luteinizing hormone; TESTO, testosterone; FSH, follicle-stimulating hormone; PROG, progesterone; E2, 17-β-estradiol; Lg (), values after log 10 transformation; SBP, systolic blood pressure; DBP, diastolic blood pressure. Bold values highlight the P value of <0.05.*

In premenopausal subjects (Supplementary Table 3), multivariate Cox proportional hazards regression analysis demonstrated that baseline E2 and FSH levels were significantly associated with the risk of POAG progression (HR = 0.04, 95% CI = 0.01 – 0.34, *p* < 0.01, and HR = 5.36, 95% CI = 1.39–20.74, *p* = 0.02, respectively). In postmenopausal subjects (Supplementary Table 4), multivariate Cox regression analysis demonstrated that baseline levels of LH (HR = 0.01, 95% CI = 0.00–0.37, *p* = 0.01) and FSH (HR = 0.03, 95% CI = 0.00–0.90, *p* = 0.04) were significantly associated with the development of POAG. We also found that the baseline level of E2 might be associated with VF progression in postmenopausal women (HR = 0.35); however, the finding was not statistically significant.

**FIGURE 2:**
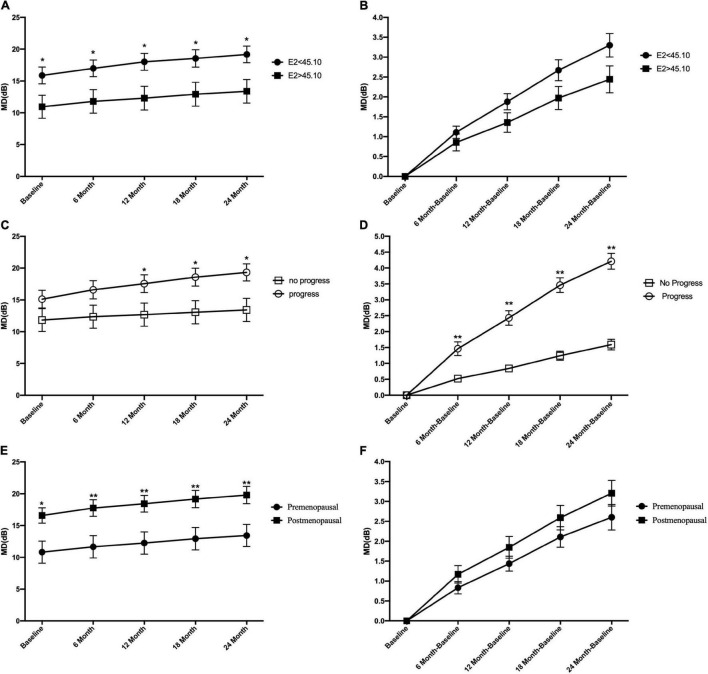
Comparison of the mean level of visual field mean deviation (MD) and mean change of visual field mean deviation between groups at the different follow-up time points. At baseline and all time points during follow-up period, the mean level of MD in E2 < 45.1 and E2 > 45.1 group **(A)**, in progression and no progression groups **(C)**, and in premenopausal and postmenopausal groups **(E)**. The mean change of MD in E2 < 45.1 and E2 > 45.1 group **(B)**, in progression and no progression groups **(D)**, in premenopausal and postmenopausal groups **(F)**. E2, 17-β-estradiol; MD, mean deviation; *p < 0.05, **p < 0.01.

Based on the median E2 level, all patients with POAG were divided into subgroups of E2 < 45.1 and E2 > 45.1. Figure 2A shows that the mean MD level at baseline and during the entire follow-up period in the E2 < 45.1 group was significantly higher than that in the E2 > 45.1 group (all *p* < 0.05). However, there were no significant differences in the mean change in MD between the E2 < 45.1 and E2 > 45.1 groups (Figure 2B). Between the progression group and the no progression group, the mean level of MD was significantly different at 12, 18, and 24 months (all *p* < 0.05), and the mean changes in MD were significantly different during the entire follow-up period (all *p* < 0.05) (Figures 2C,D). Postmenopausal subjects with POAG had significantly higher MD at baseline (*p* < 0.05) and during the entire follow-up period (all *p* < 0.01) than premenopausal patients, and the mean changes in MD showed the same trend (Figures 2E,F).

**FIGURE 3:**
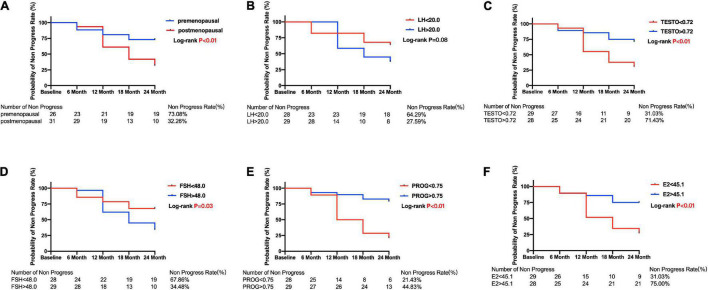
Kaplan–Meier curves for primary open angle glaucoma patients between subgroups divided by menopause or median of sex hormone level. Plots of Kaplan–Meier depicts the probability of no progression in POAG patients in each group. The number of POAG patients without progression in each group was shown below five follow-up time points. **(A)** Kaplan–Meier curves between premenopausal and postmenopausal group; Kaplan–Meier curves between lower and higher baseline level of LH **(B)**, TESTO **(C)**, FSH **(D)**, PROG **(E)**, E2 **(F)** in all POAG subjects. PRL, prolactin; LH, luteinizing hormone; TESTO, testosterone; FSH, follicle-stimulating hormone; PROG, progesterone; E2, 17-β-estradiol.

**FIGURE 4:**
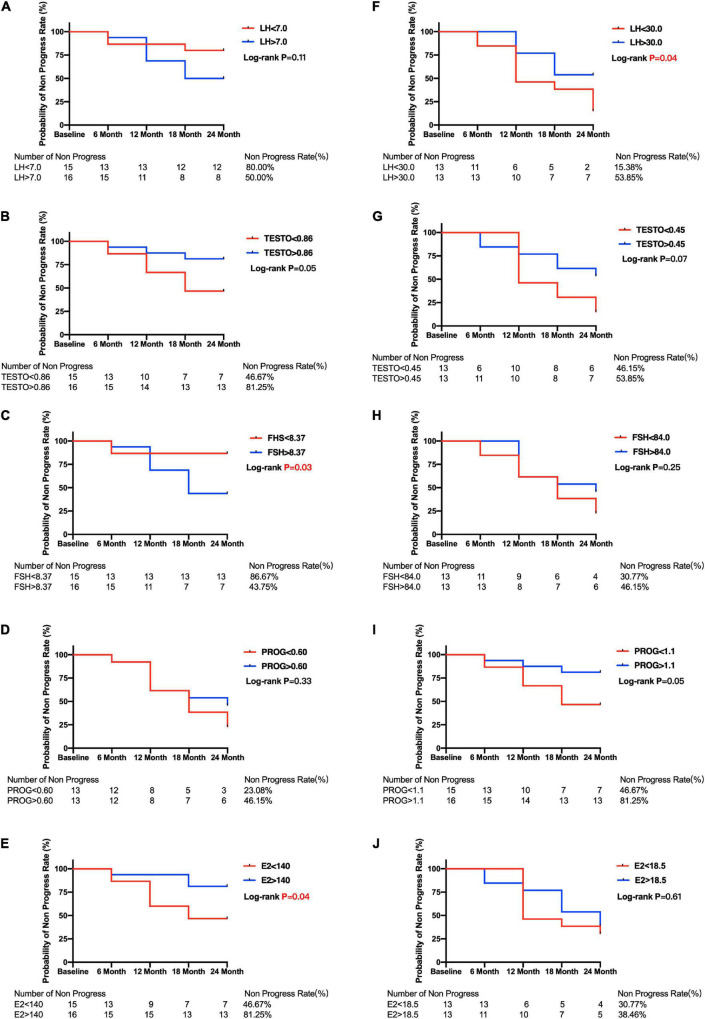
Kaplan–Meier curves for primary open angle glaucoma patients between subgroups divided by menopause or median of sex hormone level. Plots of Kaplan–Meier depicts the probability of no progression in POAG patients in each group. The number of POAG patients without progression in each group were shown below five follow-up time points. Kaplan–Meier curves between lower and higher baseline level of LH **(A)**, TESTO **(B)**, FSH **(C)**, PROG **(D)**, E2 **(E)** in premenopausal and LH **(F)**, TESTO **(G)**, FSH **(H)**, PROG **(I)**, E2 **(J)** in postmenopausal POAG subjects. PRL, prolactin; LH, luteinizing hormone; TESTO, testosterone; FSH, follicle-stimulating hormone; PROG, progesterone; E2, 17-β-estradiol.

The Kaplan–Meier survival curves are shown in Figures 3, 4. Compared with the postmenopausal patients (33.26%), the premenopausal patients had a higher non-progression rate (73.08%; log-rank test *p* < 0.01) (Figure 3A). Figure 3F shows that female patients with POAG in the E2 < 45.1 group had a lower non-progression rate (31.03%) than subjects in the E2 > 45.1 group (75.00%; log-rank test *p* < 0.01).

The median E2 level was used to divide the premenopausal and postmenopausal subjects with POAG into lower and higher baseline E2 level groups. Then, the Kaplan–Meier survival curves were generated. Figure 4E shows that premenopausal patients with lower E2 levels (46.67%) had a significantly lower non-progression rate than premenopausal patients with higher E2 levels (81.25%; log-rank test *p* = 0.04). Postmenopausal patients with lower E2 levels also had a lower non-progression rate (30.77%) than postmenopausal patients with higher E2 levels (38.48%), but the difference was not statistically significant (log-rank test *p* = 0.61) (Figure 4J). Moreover, Kaplan–Meier survival curves were generated, and log-rank analyses were performed to estimate the associations between POAG progression and the baseline sex hormone levels (PROG, FSH, LH, and TESTO) in patients with POAG, and in the premenopausal and postmenopausal subgroups (Figure 4).

## Discussion

In this work, we detected the circulation levels of sex hormones, including PRL, LH, TESTO, FSH, PROG, and E2, to assess the associations between sex hormones and POAG. In a cross-sectional study, we found that the E2 level in female patients with POAG was significantly lower than the level in control subjects, and that the decreased E2 level was a significant risk factor of POAG. The findings from the related cohort study showed that the baseline E2 level was significantly negatively correlated with VF progression. Our findings suggest that E2 is associated with glaucoma onset and progression in female patients with POAG, especially in premenopausal subjects. Moreover, we found that the circulation levels of PROG, LH, FSH, and TESTO were also significantly associated with the onset and/or development of POAG, indicating that sex hormones might jointly influence POAG pathogenesis. These findings are clinically significant in terms of their relevance to glaucoma prevention, treatment, and prognosis.

Recently, several lines of evidence have indicated that E2 influences the onset of POAG in women (Hulsman et al., 2001; Vajaranant et al., 2018). In the population-based Rotterdam Study, it was found that women who experienced menopause at <45 years of age had a significantly higher risk of POAG than women who experienced menopause at >50 years of age (Hulsman et al., 2001). In addition, a randomized trial of hormone therapy with 25,535 women and a retrospective longitudinal cohort study that enrolled 152,163 postmenopausal women suggested that hormone therapy consisting of E2 only reduced the incidence of open-angle glaucoma (Newman-Casey et al., 2014; Vajaranant et al., 2018). Other studies have suggested that decreased E2 might be associated with POAG pathogenesis. In agreement with such findings, our study evaluated the serum E2 level in POAG and normal subjects and found that decreased E2 was a risk factor of POAG. However, one case–control study that focused on the relationship between circulating E2 and POAG showed no significant associations between plasma E2 levels and POAG risk or maximum IOP (Kang et al., 2018). There are several reasons for the contradiction between the results of this case–control study and our study. First, we tested E2 levels in patients diagnosed with POAG, whereas Patel et al. (2018) tested E2 levels in individuals before they were diagnosed with POAG. What’s more, it is possible that glaucoma is a multifactorial disease, and the modest influence of E2 makes its impact difficult to confirm in the presence of other risk factors. Therefore, future trial groups must include extensive matching of age, genetic background, and other factors. The two surveys also had differences in matching risk factors and the use of multiple logistic regression models.

Furthermore, for the first time, we have shown that an increased E2 level slows the progression of VF loss in POAG. E2 is thought to play an important role in neuroprotection (Morissette et al., 2008), which has been reported in various central nervous system injuries (Groswasser et al., 1998). A study by Groswasser et al. (1998) showed that the predicted outcome and recovery were better in female patients than in male patients after traumatic brain injury. Neurodegenerative animal models, including those for Parkinson’s disease (Al Sweidi et al., 2012), Alzheimer’s disease (Uddin et al., 2020), and multiple sclerosis (Spence et al., 2013), have been used to confirm the beneficial effects of E2.

The mechanism used by E2 to prevent VF progression is unclear. Previous studies have indicated that elevated levels of E2 may decrease IOP by modifying ocular compliance and increasing the aqueous outflow facility. Multiple previous studies have confirmed that high IOP pressure accompanies decreased E2 levels (Tint et al., 2010; Patel et al., 2018). Several studies have also shown that postmenopausal women undergoing postmenopausal hormone therapy have significantly lower IOP than those who are not (Altintaş et al., 2004; Uncu et al., 2006). In addition, pregnant women were found to have reduced IOP, and pregnant women with twins have significantly greater reductions in IOP than those carrying one child because of their increased level of E2 (Saylik and Saylık, 2014). In our study, we also found that E2 is significantly negatively correlated with IOP. Our findings and those of the above study indicate that E2 might slow down the progression of VF impairment *via* decreased IOP (Green et al., 1988; Ziai et al., 1994; Feola et al., 2020).

Estrogen might also have glaucoma retinal ganglion cell (RGC)-protective effects mediated *via* a range of mechanisms. It has been reported that E2 beneficially affects vasospasm and ocular vascularization in women with POAG. Low levels of E2 in patients with POAG lead to dysfunction of autoregulation, reduced retrobulbar blood flow, and ischemic damage of the optic nerve head (Prada et al., 2016). Optic nerve degeneration is the main pathological manifestation of glaucoma. In a glaucoma model of surgically elevated IOP in rats, the number of apoptotic cells in the RGC layer was significantly decreased in the E2-treated group (Russo et al., 2008; Prokai-Tatrai et al., 2013). Several studies have found that E2 has an effect on RGC proliferation and increases RGC viability (Revankar et al., 2005; Nakazawa et al., 2006; Hayashi et al., 2007; Giordano et al., 2011). Due to its vasodilatory activity, E2 might break down the blood–retina barrier and attenuate RGC osmotic swelling (Neumann et al., 2010; Chen et al., 2011). A study indicated that E2 protects RGCs *via* inhibiting endoplasmic reticulum stress under hyperoxia (Li R. et al., 2020). E2 is also thought to protect against oxidative stress *via* an estrogen receptor (ER)-mediated mechanism (Borrás et al., 2005; Viña et al., 2006). Moreover, ERs have been found in the retina, cornea, lens, iris, ciliary body, conjunctiva, and lacrimal glands of the eyes across several species, including humans, rodents, and rabbits (Wickham et al., 2000; Munaut et al., 2001; Gupta et al., 2005). Therefore, we speculated that E2 effects glaucoma by protecting optic nerve degeneration *via* ERs by antiinflammatory (Li S. et al., 2020) and antiapoptotic means or its capability to suppress oxidative stress.

An association between lower E2 levels and risk of POAG and VF progression was observed in both premenopausal and postmenopausal subjects. However, the association was statistically significant only in the premenopausal subjects, and not in the postmenopausal subjects. Several reasons might clarify this phenomenon. First, the level of E2 in postmenopausal subjects was relatively low, and the fluctuations were relatively small because of a marked decline in the quality and quantity of the ovarian follicles (Burger et al., 2007). The level of E2 was distinctly lower in both postmenopausal POAG and normal subjects. Therefore, the polymorphisms of the E2 receptor might have a greater influence on the decreasing POAG incidence than the slightly decreased E2 levels in postmenopausal POAG subjects (de Voogd et al., 2008). Second, the sample sizes in our cross-sectional and cohort studies were not large enough after being divided into premenopausal and postmenopausal subgroups.

Except for E2, our results showed that other sex hormone levels were significantly associated with the onset and/or development of POAG. In this study, a decreased PROG level was significantly associated with POAG progression. The role of PROG in POAG subjects is still controversial. A cross-sectional study found that E2 and PROG treatment can decrease IOP in non-glaucomatous eyes (Tint et al., 2010). However, a retrospective longitudinal study found that only subjects who used E2 alone showed significant decreases in the incidence of POAG, and that there was no significant association between E2 + PROG use and POAG onset (Newman-Casey et al., 2014). Additionally, we found that the level of TESTO was significantly associated with the risk of POAG, and Kang et al. (2018) reported similar results. Furthermore, LH and FSH are secreted by the pituitary to stimulate E2 production. The decreased level of E2 in postmenopausal subjects results in altered feedback of increased FSH and LH (van den Beld et al., 2018). We found that the levels of LH and FSH were associated with POAG progression in postmenopausal subjects. We speculate that a lower level of E2 may stimulate the secretion of LH and FSH *via* a negative feedback mechanism, which is associated with POAG progression. We further hypothesize that E2 might be the original and most effective factor in POAG development. Meanwhile, there are complex interactions among sex hormones—all of which might take part in the progression of POAG. Further clinical cohort studies and basic research are needed to clarify the role of sex hormones in POAG.

In our work, we also found that age and diabetes were associated with POAG progression, which is in agreement with previous research (Song et al., 2016; Mancino et al., 2018). In addition, there was a significantly higher hypertension rate in the progression subgroup than in the no progression subgroup. The same phenomenon has been reported by others (Langman et al., 2005), as well as that IOP is a risk factor of POAG progression (Le et al., 2003; Ekström, 2012). However, in our work, there was no significant difference in IOP between the progression group and the no progression group (Table 4). The reason for this might be that many subjects had received drug treatments for IOP.

Previous studies have attempted to identify the association between the progression of glaucoma and corneal biomechanics, including CCT and corneal hysteresis (CH); there are reports that lower CCT and CH are significantly associated with a higher risk of VF deviation in glaucoma (Congdon et al., 2006; Susanna et al., 2019). However, in our study, there was no significant difference in CCT between the no progression and progression groups (*p* = 0.64) (Table 4). The reasons for this might be that the objects were different in our study and previous studies (POAG vs. glaucoma), or that the ethnic groups were different. Thus, the associations and mechanisms explaining this relationship need to be clarified by further research. Furthermore, CCT may be influenced by sex hormones. Walter et al. (2019) reported that E2 might affect the stiffness of cornea tissue, but that it did not significantly affect CCT in a porcine cornea model. Our study found that sex hormones were not associated with CCT in a Spearman analysis, univariate linear regressions, and multiple linear regressions (Supplementary Tables 5–7). Bahadir Kilavuzoglu et al. (2018) also found that E2 and PROG did not have effects on the cornea. To sum up, our study and previous studies suggest that sex hormones are not associated with CCT, but further studies are needed to confirm our results.

Although our study is the first to report the association between serum E2 and the risk of and VF progression in POAG, it has several limitations. First, it was a single-center, small sample study. All subjects enrolled in the cross-sectional and cohort studies were from the same hospital, which cannot indicate ethnic and geographical diversity in POAG pathology. Second, the patients enrolled in our study had been diagnosed with POAG. After they joined our study, we investigated and recorded their medical treatments during the follow-up period. However, we did not investigate the duration of POAG or the respective medications that the patients received before enrollment. Third, optical coherence tomography (OCT) was not performed during the follow-up period. Thus, the structural damage data obtained from OCT was not included in this study. Fourthly, the examinations of IOP and OCT were not performed in the normal group. However, on all normal subjects slit-lamp and gonioscopy were performed, and no ocular symptoms of glaucoma or family history of glaucoma was confirmed by specialty physicians. Even if few patients with POAG were included in the normal group, the bias of results was quite limited. Finally, the sample sizes of our cross-sectional and cohort studies were not large enough after dividing them into premenopausal and postmenopausal subgroups, and the results could be impacted by unknown factors. Therefore, further large-sample population-based cohort studies are warranted to measure E2 before POAG onset and to predict the risk of POAG onset and progression.

In conclusion, we have shown that a decreased level of E2 is a risk factor of POAG, and it significantly affects VF progression in women with POAG, especially in premenopausal subjects. Therefore, E2 might be a new predictor of POAG onset and VF progression, which is helpful in guiding clinical treatments and evaluating prognosis. Moreover, sex hormones, including PROG, FSH, and LH, might play a role in the pathogenesis and progression of POAG. Further prospective multicenter longitudinal studies and biological experiments are warranted to confirm our results and elucidate the underlying mechanisms.

## Data Availability Statement

The original contributions presented in the study are included in the article/Supplementary Material, further inquiries can be directed to the corresponding authors.

## Ethics Statement

The studies involving human participants were reviewed and approved by Institutional Review Board/Ethics Committee approval from the Ethics Committee of the Eye & ENT Hospital. The patients/participants provided their written informed consent to participate in this study. Written informed consent was obtained from the individual(s) for the publication of any potentially identifiable images or data included in this article.

## Author Contributions

YQ, JY, SL, WC, and YS performed the statistical analysis, drafted this article, and interpreted the data. LT, MS, and JR contributed to data collection, literature search, and statistical analysis. XS critically revised this article. SL, WC, and YS was involved in the design of this study and supervised this project. All authors contributed to the article and approved the submitted version.

## Conflict of Interest

The authors declare that the research was conducted in the absence of any commercial or financial relationships that could be construed as a potential conflict of interest.

## Publisher’s Note

All claims expressed in this article are solely those of the authors and do not necessarily represent those of their affiliated organizations, or those of the publisher, the editors and the reviewers. Any product that may be evaluated in this article, or claim that may be made by its manufacturer, is not guaranteed or endorsed by the publisher.

## Supplementary Material

Supplementary Figure 1 **(A–H)** Spearman analysis for correlation between blood levels of sex hormone and ocular parameters. In the Scatter plot, every plot means a subject. PRL, prolactin; LH, luteinizing hormone; TESTO, testosterone; FSH, follicle-stimulating hormone; PROG, progesterone; E2, 17-β-estradiol; IOP, intraocular pressure; ACD, anterior chamber depth; MD, mean deviation.
