# Supplementary figures and images for: Association Between Sex Hormones and Visual Field Progression in Women With Primary Open Angle Glaucoma: A Cross-Sectional and Prospective Cohort Study
Source: Front Aging Neurosci. 2021 Dec 24;13:756186. doi: 10.3389/fnagi.2021.756186 (PMC8741302; doi:10.3389/fnagi.2021.756186)

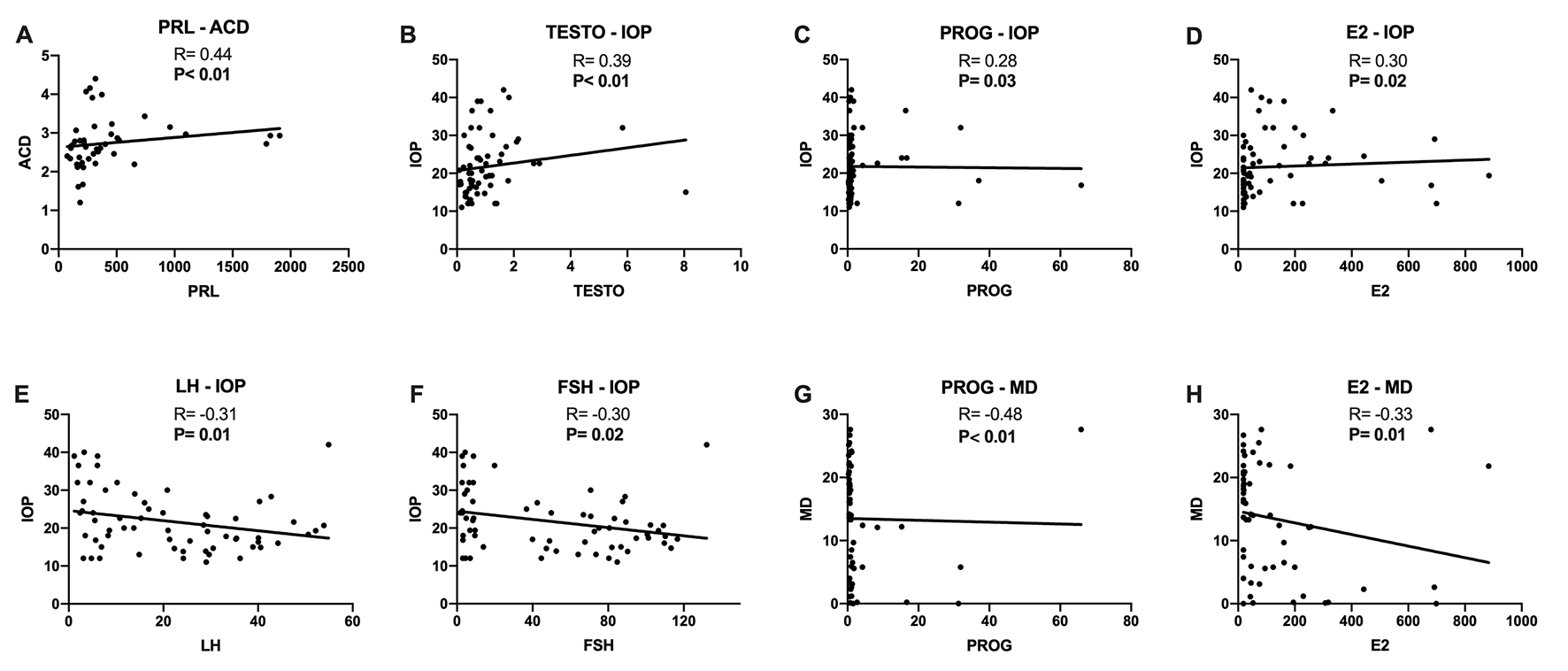

Supplement: Supplementary Figure 1 — (A–H) Spearman analysis for correlation between blood levels of sex hormone and ocular parameters. In the Scatter plot, every plot means a subject. PRL, prolactin; LH, luteinizing hormone; TESTO, testosterone; FSH, follicle-stimulating hormone; PROG, progesterone; E2, 17-β-estradiol; IOP, intraocular pressure; ACD, anterior chamber depth; MD, mean deviation. [file Image_1.TIFF]
