# Supplementary material for: Association Between Sex Hormones and Visual Field Progression in Women With Primary Open Angle Glaucoma: A Cross-Sectional and Prospective Cohort Study
Source: Front Aging Neurosci. 2021 Dec 24;13:756186. doi: 10.3389/fnagi.2021.756186 (PMC8741302; doi:10.3389/fnagi.2021.756186)
Supplement: Supplementary file 2 [file Table_1.DOCX]

Supplemental table 1. Univariate and Multivariate Logistic Regression Analyses to Identify Risk Factors for Premenopausal Women with POAG

|  | Univariate | |  | Multivariate | |
| --- | --- | --- | --- | --- | --- |
|  | P value | OR (95%CI) |  | P value | OR (95%CI) |
| Age | **0.049** | 0.94 (0.88 to 1.00) |  |  | |
| Diabetes | 1.00 | 0.00 (0.00 to 0.00) |  |  |  |
| Hypertension | 0.23 | 4.00 (0.42 to 37.84) |  |  |  |
| BMI | 0.42 | 0.94 (0.80 to 1.10) |  |  |  |
| PRL | 0.79 | 1.00 (0.99 to 1.00) |  | 0.98 | 1.00 (1.00 to 1.00) |
| LH | 0.55 | 0.99 (0.96 to1.03) |  | 0.96 | 1.00 (0.96 to 1.04) |
| TESTO | 0.13 | 1.98 (0.82 to 4.78) |  | 0.34 | 1.46 (0.67 to 3.17) |
| FSH | 0.54 | 1.01 (0.99 to 1.02) |  | 0.23 | 1.01 (0.99 to 1.04) |
| PROG | 0.37 | 0.99 (0.96 to 1.02) |  | 0.41 | 0.99 (0.95 to 1.02) |
| E2 | **0.03** | 0.99 (0.99 to 1.00) |  | **0.03** | 1.00 (0.99 to 1.00) |
| Lg (PRL) | 0.26 | 0.37 (0.07to 2.07) |  | 0.42 | 0.48 (0.08 to 2.87) |
| Lg (LH) | 0.32 | 0.51 (0.13 to 1.94) |  | 0.98 | 0.98 (0.22 to 4.46) |
| Lg (TESTO) | **0.046** | 8.79 (1.04 to 74.39) |  | 0.63 | 1.68 (0.21 to 13.64) |
| Lg (FSH) | 0.65 | 0.77 (0.25 to 2.37) |  | 0.27 | 2.25 (0.53 to 9.51) |
| Lg (PROG) | 0.80 | 0.92 (0.45 to 1.83) |  | 0.56 | 0.79 (0.36 to 1.73) |
| Lg (E2) | **0.03** | 0.30 (0.10 to 0.88) |  | **<0.01** | 0.09 (0.02 to 0.48) |

POAG = primary open angle glaucoma, OR = odds ratio, CI = confidence interval, BMI =body mass index, PRL = prolactin, LH = luteinizing hormone, TESTO = testosterone, FSH = follicle-stimulating hormone, PROG = Progesterone, E2 =17- 𝛃 -estradiol, Lg( ) = values after log 10 transformation. Multivariate logistic regression was adjusted for age, BMI, diabetes (yes = 1, no = 0), hypertension (yes = 1, no = 0).

Supplemental table 2. Univariate and Multivariate Logistic Regression Analyses to Identify Risk Factors for Postmenopausal Women with POAG

|  | Univariate | |  | Multivariate | |
| --- | --- | --- | --- | --- | --- |
|  | P value | OR (95%CI) |  | P value | OR (95%CI) |
| Age | 0.10 | 1.12 (1.02 to 1.20) |  |  | |
| Diabetes | 0.65 | 1.77 (0.15 to 20.82) |  |  |  |
| Hypertension | 0.55 | 1.40 (0.47 to 4.20) |  |  |  |
| BMI | 0.08 | 0.81 (0.65 to 1.02) |  |  |  |
| PRL | 0.87 | 1.00 (0.99 to 1.01) |  | 0.99 | 1.00 (0.99 to 1.01) |
| LH | 0.76 | 1.01 (0.96 to 1.06) |  | 0.86 | 1.01 (0.95 to 1.06) |
| TESTO | 0.25 | 2.28 (0.55 to 9.43) |  | **0.02** | 10.50 (1.41 to 78.51) |
| FSH | **0.02** | 1.03 (1.00 to 1.06) |  | 0.09 | 1.02 (1.00 to 1.05) |
| PROG | **0.02** | 0.14 (0.03 to 0.74) |  | 0.07 | 0.18 (0.03 to 1.12) |
| E2 | 0.33 | 1.97 (0.95 to 1.02) |  | 0.96 | 1.00 (0.96 to 1.04) |
| Lg (PRL) | 0.84 | 1.37 (0.07 to 25.98) |  | 0.88 | 1.31 (0.05 to 37.09) |
| Lg (LH) | 0.92 | 1.19 (0.05 to 29.46) |  | 0.81 | 0.66 (0.02 to 20.61) |
| Lg (TESTO) | 0.31 | 2.13 (0.50 to 9.03) |  | **0.03** | 9.89 (1.26 to 77.65) |
| Lg (FSH) | 0.09 | 22.54 (0.60 to 845.32) |  | 0.31 | 7.24 (0.15 to 341.66) |
| Lg (PROG) | **0.01** | 0.01 (0.00 to 0.31) |  | **0.04** | 0.02 (0.00 to 0.75) |
| Lg (E2) | 0.28 | 0.21 (0.01 to 3.56) |  | 0.91 | 0.83 (0.04 to 18.68) |

POAG = primary open angle glaucoma, OR = odds ratio, CI = confidence interval, BMI =body mass index, PRL = prolactin, LH = luteinizing hormone, TESTO = testosterone, FSH = follicle-stimulating hormone, PROG = Progesterone, E2 =17- 𝛃 -estradiol, Lg( ) = values after log 10 transformation. Multivariate logistic regression was adjusted for age, BMI, diabetes (yes = 1, no = 0), hypertension (yes = 1, no = 0).

Supplemental table 3. Univariate Cox Proportional Hazards Regression Analysis and Multivariate Cox Proportional Hazards Regression Analysis to Assess the Value of Baseline Parameters Associated with Progression of POAG in Premenopausal Women.

|  | Univariate | | Multivariate | |
| --- | --- | --- | --- | --- |
|  | P | HR (95%CI) | P | HR (95%CI) |
| Age | 0.41 | 1.02 (0.97 to 1.09) |  |  |
| Diabetes | **0.01** | 19.66 (1.94 to 198.84) |  |  |
| Hypertension | 0.13 | 2.76 (0.73 to 10.43) |  |  |
| BMI | 0.62 | 1.04 (0.89 to 1.23) |  |  |
| SBP | 0.88 | 1.01 (0.94 to 1.08) |  |  |
| DBP | 0.99 | 1.00 (0.96 to 1.04) |  |  |
| PRL | 0.56 | 1.00 (1.00 to 1.00) | 0.87 | 1.00 (1.00 to 1.00) |
| LH | 0.18 | 1.03 (0.99 to 1.07) | 0.14 | 1.04 (0.99 to 1.09) |
| TESTO | 0.06 | 0.22 (0.05 to 1.07) | 0.06 | 0.10 (0.01 to 1.15) |
| FSH | **0.02** | 1.02 (1.00 to 1.03) | **0.03** | 1.02 (1.00 to 1.04) |
| PROG | 0.20 | 0.46 (0.14 to 1.52) | 0.20 | 0.50 (0.17 to 1.46) |
| E2 | **0.04** | 0.99 (0.98 to 1.00) | **<0.01** | 0.99 (0.98 to 1.00) |
| Lg (PRL) | 0.89 | 1.13 (0.22 to 5.73) | 0.77 | 0.74 (0.10 to 5.65) |
| Lg (LH) | 0.19 | 2.85 (0.60 to 13.64) | 0.22 | 3.14 (0.50 to 19.78) |
| Lg (TESTO) | **0.03** | 0.06 (0.00 to 0.72) | **0.03** | 0.01 (0.00 to 0.70) |
| Lg (FSH) | **0.01** | 4.02 (1.35 to 11.97) | **0.02** | 5.36 (1.39 to 20.74) |
| Lg (PROG) | **0.02** | 0.06 (0.01 to 0.66) | **0.03** | 0.05 (0.00 to 0.78) |
| Lg (E2) | **<0.01** | 0.17 (0.04 to 0.64) | **<0.01** | 0.04 (0.01 to 0.34) |

POAG = primary open angle glaucoma, HR = hazard ratio, CI = confidence interval, BMI =body mass index, PRL = prolactin, LH = luteinizing hormone, TESTO = testosterone, FSH = follicle-stimulating hormone, PROG = Progesterone, E2 =17- 𝛃 -estradiol, Lg( ) = values after log 10 transformation, SBP = systolic blood pressure; DBP = diastolic blood pressure. Multivariate Cox regression was adjusted for age, BMI, DBP, SBP, diabetes (yes = 1, no = 0), hypertension (yes = 1, no = 0).

Supplemental table 4. Univariate Cox Proportional Hazards Regression Analysis and Multivariate Cox Proportional Hazards Regression Analysis to Assess the Value of Baseline Parameters Associated with Progression of POAG in Postmenopausal Women.

|  | Univariate | | Multivariate | |
| --- | --- | --- | --- | --- |
|  | P | HR (95%CI) | P | HR (95%CI) |
| Age | 0.54 | 1.02 (0.96 to 1.08) |  |  |
| Diabetes | 0.19 | 2.80 (0.59 to 13.17) |  |  |
| Hypertension | 0.60 | 0.77 (0.29 to 2.03) |  |  |
| BMI | 0.11 | 0.86 (0.71 to 1.04) |  |  |
| SBP | 0.13 | 1.05 (0.99 to 1.11) |  |  |
| DBP | 0.56 | 0.99 (0.96 to 1.02) |  |  |
| PRL | 0.34 | 1.00 (1.00 to1.01) | 0.41 | 1.00 (1.00 to 1.01) |
| LH | **0.03** | 0.94 (0.89 to 0.99) | **0.04** | 0.94 (0.89 to 1.00) |
| TESTO | 0.50 | 1.18 (0.73 to 1.91) | 0.20 | 1.43 (0.83 to 2.48) |
| FSH | **0.02** | 0.98 (0.96 to 1.00) | 0.06 | 0.98 (0.96 to 1.00) |
| PROG | 0.16 | 0.22 (0.03 to 1.82) | 0.57 | 0.45 (0.03 to 6.88) |
| E2 | 0.38 | 0.98 (0.95 to 1.02) | 0.84 | 1.00 (0.95 to 1.05) |
| Lg (PRL) | 0.49 | 2.69 (0.17 to 42.88) | 0.56 | 2.46 (0.12 to 50.35) |
| Lg (LH) | **0.01** | 0.01 (0.00 to 0.25) | **0.01** | 0.01 (0.00 to 0.37) |
| Lg (TESTO) | 0.15 | 0.37 (0.10 to 1.45) | 0.43 | 0.38 (0.03 to 4.29) |
| Lg (FSH) | **<0.01** | 0.03 (0.00 to 0.37) | **0.04** | 0.03 (0.00 to 0.90) |
| Lg (PROG) | 0.25 | 0.18 (0.01 to 3.30) | 0.65 | 0.42 (0.01 to 17.64) |
| Lg (E2) | 0.27 | 0.17 (0.01 to 3.93) | 0.57 | 0.35 (0.01 to 13.34) |

POAG = primary open angle glaucoma, HR = hazard ratio, CI = confidence interval, BMI =body mass index, PRL = prolactin, LH = luteinizing hormone, TESTO = testosterone, FSH = follicle-stimulating hormone, PROG = Progesterone, E2 =17- 𝛃 -estradiol, Lg( ) = values after log 10 transformation, SBP = systolic blood pressure; DBP = diastolic blood pressure. Multivariate Cox regression was adjusted for age, BMI, DBP, SBP, diabetes (yes = 1, no = 0), hypertension (yes = 1, no = 0).

Supplemental table 5. Univariate linear regressions and Multiple Linear Regressions for Associations Between Blood Sex Hormone Level and Central Cornea Thickness in Women with POAG

| Variable | B | t Value | p Value | 95%CI |
| --- | --- | --- | --- | --- |
| Model A |  |  |  |  |
| PRL | 0.08 | 0.56 | 0.58 | -0.02 to 0.03 |
| LH | -0.12 | -0.86 | 0.40 | -1.02 to 0.41 |
| TESTO | -0.03 | -0.17 | 0.86 | -8.80 to 7.39 |
| FSH | -0.11 | -0.76 | 0.45 | -0.37 to 0.17 |
| PROG | -0.10 | -0.73 | 0.47 | -1.37 to 0.64 |
| E2 | 0.02 | 0.15 | 0.88 | -0.06 to 0.06 |
| Lg (PRL) | 0.15 | 1.04 | 0.30 | -16.15 to 51.05 |
| Lg (LH) | -0.16 | -1.15 | 0.26 | -40.38 to 11.04 |
| Lg (TESTO) | 0.002 | 0.02 | 0.99 | -27.80 to 28.29 |
| Lg (FSH) | -0.09 | -0.63 | 0.53 | -24.59 to 12.83 |
| Lg (PROG) | -0.15 | -1.08 | 0.29 | -32.02 to 9.62 |
| Lg (E2) | 0.05 | 0.37 | 0.71 | -17.56 to 25.51 |
| Model B |  |  |  |  |
| PRL | 0.08 | 0.49 | 0.63 | -0.02 to 0.04 |
| LH | -0.10 | -0.59 | 0.56 | -1.14 to 0.63 |
| TESTO | -0.04 | -0.23 | 0.82 | -9.73 to 7.73 |
| FSH | -0.07 | -0.38 | 0.70 | -0.45 to 0.30 |
| PROG | -0.15 | -1.03 | 0.31 | -1.61 to 0.52 |
| E2 | -0.09 | -0.53 | 0.60 | -0.09 to 0.05 |
| Lg (PRL) | 0.09 | 0.57 | 0.57 | -28.32 to 50.48 |
| Lg (LH) | -0.21 | -1.20 | 0.34 | -52.54 to 13.30 |
| Lg (TESTO) | 0.003 | 0.02 | 0.98 | -34.94 to 35.50 |
| Lg (FSH) | -0.05 | 0.25 | 0.80 | -31.24 to 24.36 |
| Lg (PROG) | -0.24 | -1.54 | 0.13 | -41.04 to 5.43 |
| Lg (E2) | -0.05 | -0.24 | 0.81 | -36.72 to 28.85 |

POAG = primary open angle glaucoma, B = regression coefficient, CI = confidence interval, BMI =body mass index, PRL = prolactin, LH = luteinizing hormone, TESTO = testosterone, FSH = follicle-stimulating hormone, PROG = Progesterone, E2 = 17- 𝛃 -estradiol, Lg( ) = values after log 10 transformation, SBP = systolic blood pressure, DBP = diastolic blood pressure. Model A is not adjusted. Model B adjusted for age, BMI, SBP, DBP, diabetes (yes = 1, no = 0), hypertension (yes = 1, no = 0).

Supplemental table 6. Univariate linear regressions and Multiple Linear Regressions for Associations Between Blood Sex Hormone Level and Central Cornea Thickness in Premenopausal Women with POAG

| Variable | B | t Value | p Value | 95%CI |
| --- | --- | --- | --- | --- |
| Model A |  |  |  |  |
| PRL | 0.06 | 0.28 | 0.78 | -0.03 to 0.04 |
| LH | 0.01 | 0.06 | 0.96 | -1.40 to 1.48 |
| TESTO | -0.39 | -2.09 | **0.047** | -30.25 to -0.21 |
| FSH | 0.05 | 0.24 | 0.82 | -0.48 to 0.60 |
| PROG | -0.16 | -0.82 | 0.42 | -1.72 to 0.74 |
| E2 | -0.03 | -0.15 | 0.88 | -0.09 to 0.07 |
| Lg (PRL) | 0.12 | 0.61 | 0.55 | -34.26 to 63.17 |
| Lg (LH) | -0.09 | -0.46 | 0.65 | -53.35 to 33.85 |
| Lg (TESTO) | -0.24 | -1.24 | 0.23 | -98.65 to 24.41 |
| Lg (FSH) | 0.04 | 0.18 | 0.86 | -29.42 to 35.15 |
| Lg (PROG) | -0.29 | -1.49 | 0.15 | -47.93 to 7.68 |
| Lg (E2) | -0.03 | -0.13 | 0.90 | -39.44 to -34.91 |
| Model B |  |  |  |  |
| PRL | 0.25 | 0.74 | 0.47 | -0.04 to 0.08 |
| LH | -0.002 | -0.01 | 0.99 | -1.67 to 1.65 |
| TESTO | -0.41 | -1.94 | 0.07 | -33.70 to 1.29 |
| FSH | 0.08 | 0.30 | 0.76 | -0.59 to 0.79 |
| PROG | -0.23 | -0.90 | 0.38 | -2.27 to 0.90 |
| E2 | -0.09 | -0.36 | 0.72 | -0.13 to 0.09 |
| Lg (PRL) | 0.21 | 0.74 | 0.47 | -45.15 to 94.69 |
| Lg (LH) | -0.15 | -0.63 | 0.54 | -67.30 to 36.15 |
| Lg (TESTO) | -0.38 | -1.56 | 0.14 | -136.80 to 19.96 |
| Lg (FSH) | 0.04 | 0.16 | 0.87 | -39.11 to 45.61 |
| Lg (PROG) | -0.34 | -1.44 | 0.17 | -58.17 to 10.78 |
| Lg (E2) | -0.05 | -0.20 | 0.85 | -56.48to 46.74 |

POAG = primary open angle glaucoma, B = regression coefficient, CI = confidence interval, BMI =body mass index, PRL = prolactin, LH = luteinizing hormone, TESTO = testosterone, FSH = follicle-stimulating hormone, PROG = Progesterone, E2 = 17- 𝛃 -estradiol, Lg( ) = values after log 10 transformation, SBP = systolic blood pressure, DBP = diastolic blood pressure. Model A is not adjusted. Model B adjusted for age, BMI, DBP, diabetes (yes = 1, no = 0), hypertension (yes = 1, no = 0).

Supplemental table 7. Univariate linear regressions and Multiple Linear Regressions for Associations Between Blood Sex Hormone Level and Central Cornea Thickness in Postmenopausal Women with POAG

| Variable | B | t Value | p Value | 95%CI |
| --- | --- | --- | --- | --- |
| Model A |  |  |  |  |
| PRL | 0.07 | 0.32 | 0.75 | -0.13 to 0.18 |
| LH | -0.26 | -1.28 | 0.21 | -1.90 to 0.45 |
| TESTO | 0.34 | 1.71 | 0.10 | -1.43 to 15.05 |
| FSH | -0.29 | -1.44 | 0.16 | -0.83 to 0.15 |
| PROG | 0.08 | 0.39 | 0.70 | -44.53 to 65.43 |
| E2 | 0.11 | 0.53 | 0.60 | -0.58 to 0.99 |
| Lg (PRL) | 0.14 | 0.66 | 0.52 | -49.30 to 95.49 |
| Lg (LH) | -0.31 | -1.56 | 0.13 | -120.69 to 16.79 |
| Lg (TESTO) | 0.18 | 0.85 | 0.40 | -18.16 to 43.62 |
| Lg (FSH) | -0.40 | -2.08 | **0.049** | -126.47 to -0.44 |
| Lg (PROG) | 0.11 | 0.52 | 0.61 | -64.91 to 107.98 |
| Lg (E2) | 0.07 | 0.35 | 0.73 | -55.78 to 78.52 |
| Model B |  |  |  |  |
| PRL | -0.33 | -1.85 | 0.08 | -0.25 to 0.02 |
| LH | -0.28 | -1.76 | 0.10 | -1.70 to 0.16 |
| TESTO | 0.26 | 1.20 | 0.25 | -4.07 to 14.80 |
| FSH | -0.32 | -1.99 | 0.06 | -0.79 to 0.02 |
| PROG | 0.14 | 0.63 | 0.53 | -40.34 to 75.01 |
| E2 | 0.23 | 1.26 | 0.22 | -0.28 to 1.12 |
| Lg (PRL) | -0.25 | -1.39 | 0.18 | -108.06 to 22.07 |
| Lg (LH) | -0.28 | -1.74 | 0.10 | -102.73 to 9.84 |
| Lg (TESTO) | 0.36 | 1.44 | 0.17 | -12.33 to 64.98 |
| Lg (FSH) | -0.36 | -2.20 | **0.04** | -113.07 to -2.30 |
| Lg (PROG) | 0.12 | 0.57 | 0.57 | -62.07 to 108.44 |
| Lg (E2) | 0.19 | 1.04 | 0.31 | -30.63 to 90.60 |

POAG = primary open angle glaucoma, B = regression coefficient, CI = confidence interval, BMI =body mass index, PRL = prolactin, LH = luteinizing hormone, TESTO = testosterone, FSH = follicle-stimulating hormone, PROG = Progesterone, E2 = 17- 𝛃 -estradiol, Lg( ) = values after log 10 transformation, SBP = systolic blood pressure, DBP = diastolic blood pressure. Model A is not adjusted. Model B adjusted for age, BMI, DBP, diabetes (yes = 1, no = 0), hypertension (yes = 1, no = 0).
